# Study on surface integrity modulation of 1Cr12Ni3Mo2VN steel by ultrasonic impact treatment and its inhibition mechanism against water erosion damage^☆^

**DOI:** 10.1016/j.ultsonch.2026.107959

**Published:** 2026-07-14

**Authors:** Tong Ran, Peihan Lin, Fei Sun, Yongqing Lai, Yiming Lin, Yu Zhang, Yanyu Wang, Bicheng Guo, Shiqi Chen, Qingshan Jiang

**Affiliations:** a College of Marine Equipment and Mechanical Engineering, Jimei University, Xiamen 361000, China; b Engineering Research Center of Anti-Fatigue Manufacturing for Marine Equipment (Fujian Province), Xiamen 361000, China

**Keywords:** Ultrasonic impact treatment, 1Cr12Ni3Mo2VN steel, Residual compressive stress, Surface roughness, Water erosion

## Abstract

During service, last-stage steam turbine blades are subjected to repeated high-velocity droplet impacts, leading to severe water erosion and a marked reduction in service life and reliability. In this work, a combined methodology integrating theoretical analysis, finite element simulation, and experimental validation was employed to investigate the effect of ultrasonic impact treatment (UIT) on the surface integrity and water erosion resistance of 1Cr12Ni3Mo2VN stainless steel. An axisymmetric finite element model was developed to analyze the roles of vibration amplitude, static load, and coverage rate in regulating the residual compressive stress(RCS) field. The results show that vibration amplitude dominantly controls both the magnitude and depth of RCS, while static load mainly governs the onset of plastic deformation with a negligible influence on the final stress distribution; coverage rate has a limited effect on RCS but significantly affects surface roughness, thereby influencing erosion resistance. Under the optimal parameters of 300 N static load, 10 μm amplitude, and 0.1 mm impact spacing, the maximum surface RCS reaches 1081 ± 67 MPa, accompanied by a 41.23% reduction in mass loss. The enhanced water erosion resistance is attributed to a synergistic mechanism in which reduced surface roughness and surface energy suppress cavitation bubble nucleation and growth, while the high-amplitude RCS elevates the material damage threshold.

## Graphical abstract

**Figure:**
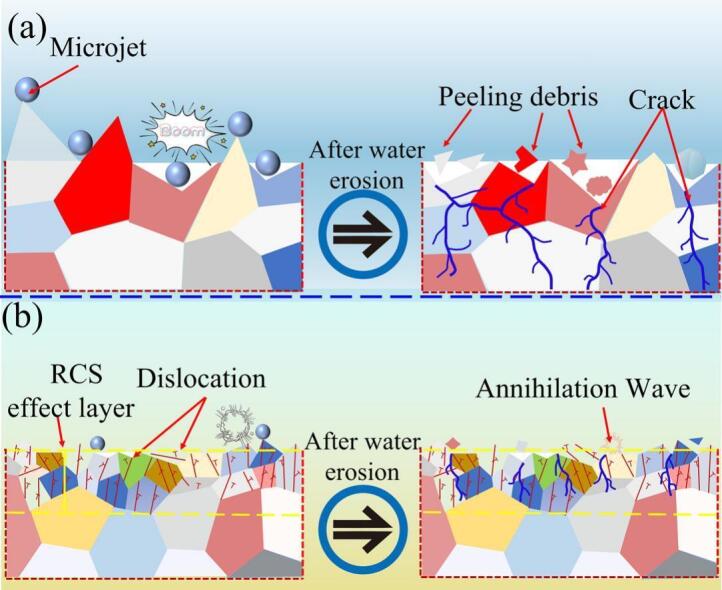

## 1 Introduction

**Fig. 1:**
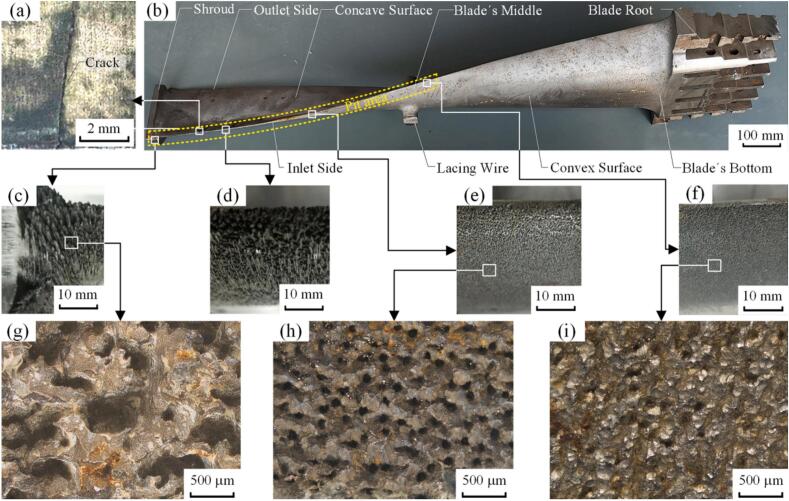
WE damage morphology of the last-stage blade of a steam turbine[10].

In thermal and nuclear power units, 1Cr12Ni3Mo2VN martensitic stainless steel is widely used for manufacturing last-stage blades due to its favorable combination of strength and toughness [1], [2]. During service, these blades operate in a wet steam environment where steam undergoes condensation during expansion, generating a large number of high-velocity droplets. Under high rotational speeds, these droplets continuously impinge on the blade surface, leading to a typical form of surface degradation known as water erosion (WE). WE is a dynamic damage process induced by repetitive high-speed droplet impacts [3], [4], generally characterized by four stages: incubation, acceleration, deceleration, and steady-state. During the incubation stage, plastic deformation accumulates within the near-surface layer without significant mass loss; as the number of impacts increases, microcracks initiate and progressively propagate under cyclic loading, ultimately resulting in material removal and pit formation [5], [6]. Throughout this process, droplet impacts generate localized transient high pressures and stress waves, with peak stresses often exceeding the yield strength of the material. Moreover, cavitation effects accompanying droplet impact further aggravate material degradation, as the collapse of WE bubbles produces microjets and impact waves that impose severe localized damage on the material surface [7], [8], [9],the structure is shown in Fig. 1.

Early studies on WE primarily focused on the influence of intrinsic mechanical properties of materials. Young [11] proposed that material hardness is positively correlated with erosion resistance, indicating that harder materials generally exhibit superior resistance to impact-induced damage. Hattori and Lin [12] investigated the effects of droplet size and impact velocity, demonstrating that WE behavior is governed not only by material properties but also by external loading conditions. In recent years, increasing attention has been paid to the role of surface integrity and material structure organization in erosion resistance. Di [13] and Kirols [14] reported that reduced surface roughness effectively mitigates stress concentration, thereby delaying crack initiation and improving erosion resistance.Lassi et al [15] demonstrated that quenching and partitioning heat treatment can significantly improve the cavitation erosion resistance of AISI 420-type martensitic stainless steel. The enhanced resistance was mainly attributed to the synergistic effect of high initial hardness and a certain amount of retained austenite. During cavitation erosion, the retained austenite absorbs impact energy through strain-induced martensitic transformation, while the accompanying lattice expansion helps suppress the propagation of intergranular cracks. Li et al. [16] further showed that alloying is also an effective strategy for improving the cavitation erosion resistance of stainless steels. The addition of Mo to 316 L stainless steel was found to significantly enhance its cavitation erosion resistance in a 3.5% NaCl solution.

To enhance WE resistance, various surface modification techniques have been proposed,Mordyuk et al [17] systematically reviewed the application of various surface-treatment technologies for improving the cavitation erosion resistance of metallic components, including micro-arc oxidation, high-velocity oxy-fuel spraying, cold spraying, laser surface alloying, nitriding, friction stir processing, and tungsten inert gas remelting. The review covered Fe-, Cu-, Al-, Ni-, and Ti-based alloy systems and reported that the cavitation erosion resistance of coatings and modified surface layers can be approximately 6–30 times higher than that of untreated materials.Gujba [18] demonstrated that WC-based coatings fabricated by high-velocity air fuel spraying exhibit excellent WE resistance.Meanwhile, Rahul [19] reported that the carbide-reinforced layer formed during electrical discharge machining can significantly improve surface performance and erosion resistance. However, these methods still present limitations in engineering applications: thermal spray coatings are prone to delamination under cyclic loading, while electrical discharge machining, despite improving hardness, often increases surface roughness, which may adversely affect erosion resistance. Therefore, it is necessary to develop a surface strengthening method that combines low cost, high efficiency, and excellent surface integrity.

In this context, ultrasonic impact treatment (UIT) has attracted increasing attention as an emerging surface modification technique [20], [21], [22]. Mordyuk and Prokopenko [23] reported that UIT can significantly improve fatigue performance by optimizing surface microstructure and stress states, meanwhile, Zaporozhets et al [24], found that the crystallographic texture induced by severe plastic deformation in the surface layer during ultrasonic impact treatment is an important factor affecting material hardness and service performance. The region exhibiting the strongest texture corresponded to the area with the highest strain and hardness. Dang [25] further showed that ultrasonic rolling can effectively reduce surface roughness and generate gradient nanostructures, thereby enhancing wear resistance. UIT induces severe plastic deformation in the surface layer through the superposition of high-frequency mechanical vibration on static loading, leading to grain refinement, work hardening, and the introduction of RCS [22], [26]. The induced RCS can offset the tensile stress generated during droplet impact, thereby suppressing crack initiation and propagation. Cao [27] further indicated that RCS interacts synergistically with phase composition to influence erosion resistance. Zhao [28] emphasized that the synergy between RCS and gradient microstructures is a key mechanism for improving mechanical performance. Xu and Lesyk [29], [30] found that severe plastic deformation induced by UIT generates high-density dislocation structures that evolve into refined grains or even nanocrystalline structures, significantly enhancing strength and impact resistance.Wu et al. [31] found that ultrasonic surface rolling processing (USRP) significantly refined the grains and eliminated surface defects, thereby improving cavitation erosion resistance. However, when the rolling-induced plastic deformation exceeded a certain level, the grain-refinement effect weakened and rolling-fatigue-related surface defects developed, leading to a sharp deterioration in cavitation erosion resistance. These results indicate that USRP has an optimal processing-parameter window. Although UIT has achieved remarkable progress in improving fatigue and wear resistance [32], [33], these performance improvements are generally associated with the combined effects of surface hardening, grain refinement, gradient microstructure formation, and RCS. However, the individual roles of the main UIT processing parameters, including static load, vibration amplitude, and impact coverage, in regulating surface roughness, the magnitude and depth of RCS, and the subsequent WE behavior have not yet been sufficiently distinguished or systematically discussed.

Unlike previous studies that mainly focused on the overall strengthening effects or microstructural evolution induced by ultrasonic surface treatment, the present work combines theoretical analysis, finite element simulation, and experimental validation to establish the relationship between processing parameters and WE resistance for 1Cr12Ni3Mo2VN martensitic stainless steel. The main contributions of this study are threefold. First, the respective effects of static load, vibration amplitude, and impact coverage on the residual compressive stress field and surface roughness are quantitatively distinguished. Second, the effective spatial range of residual-stress superposition is correlated with the diameter of a single impact dimple, providing a basis for selecting an appropriate impact spacing. Finally, a stage-dependent barrier–synergy framework is proposed. During the incubation stage of WE, surface smoothing mainly reduces the tendency for bubble attachment and heterogeneous nucleation. During the subsequent erosion stage, the subsurface RCS field primarily delays impact-induced crack initiation and propagation.

## 2 Simulation modeling and theoretical analysis of UIT

### 2.1 Model establishment and parameter selection

An axisymmetric UIT model consisting of the horn, impact head, and workpiece was established, and the dynamic implicit analysis method was employed to investigate the transient response characteristics during the impact process. The material behavior was described using the Johnson–Cook constitutive model, as expressed in Eq.(1), and the corresponding J–C parameters for 1Cr12Ni3Mo2VN stainless steel are listed in Table 1.

(1) $$\sigma =\text{(A}+\text{B}\varepsilon^{n}\text{)(1}+\text{Cln}\frac{\dot{\varepsilon }}{{\dot{\varepsilon }}_{\text{0}}}\text{)(1 -}\;{\left[\frac{T-T_{r}}{T_{m}-T_{r}}\right]}^{m})$$

**Table 1:** Model parameter table of 1Cr12Ni3Mo2VN steel.

Material	A(MPa)	B(MPa)	*n*	C	*m*
1Cr12Ni3Mo2VN	1060	621	0.229	0.01	1

**Fig. 2:**
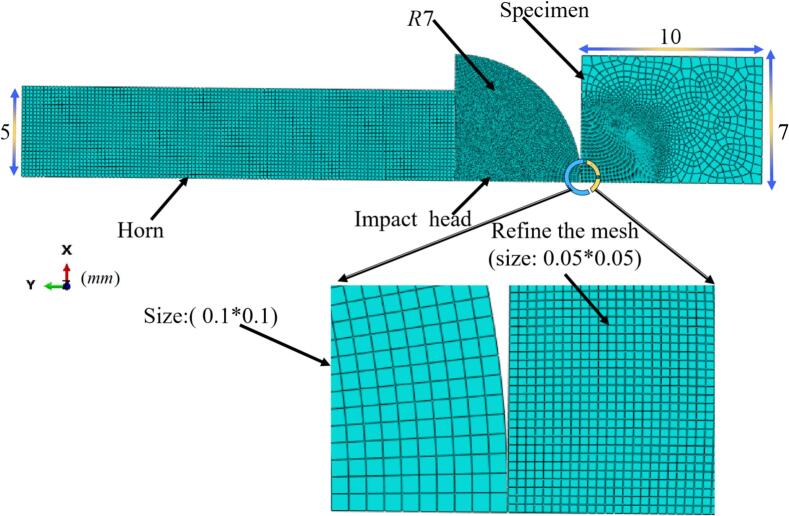
Finite element simulation modeling.

An axisymmetric UIT simulation model was established using ABAQUS, as shown in Fig. 2. To replicate the actual ultrasonic processing conditions, a pressure boundary was first applied to the front end of the horn to represent the static load, followed by a sinusoidal displacement excitation with a frequency of 27 kHz imposed at the rear end of the horn, generating a harmonic response with a displacement amplitude of *A* μm. In this manner, both the static load and vibration amplitude during the UIT were effectively implemented. Regarding boundary conditions, the bottom surface of the specimen was fully constrained, while the symmetry axis was assigned an XSYMM axisymmetric boundary condition to ensure model consistency.

To systematically investigate the role of static load in the UIT, the applied static load was categorized into two distinct regimes: (1) a sub-yield stage, in which the static load does not exceed the material yield strength and the material remains in the elastic state prior to UIT loading; (2) a super-yield stage, in which the static load exceeds the yield strength, causing the material to enter the plastic deformation regime even before the application of.

Based on Hertzian contact theory [34], given that the radius of the impacting sphere is *R* = 7 *mm*, the initial yield strength of the material is A *=* 1060 MPa, the elastic modulus of the workpiece is *E*_1_ = 210 GPa, he elastic modulus of the impacting sphere is *E*_2_ = 520 GPa, and the Poisson’s ratio for both materials is *ν* = 0.3, the equivalent elastic modulus E* can be calculated according to Eq.(2):

(2) $$E*={\left(\frac{1-v_{1}^{2}}{E_{1}}+\frac{1-v_{2}^{2}}{E_{2}}\right)}^{-1}$$

Substituting the material parameters into Eq.(2), the equivalent elastic modulus is calculated as E*=164.4 GPa. According to Eq.(3), when the maximum contact pressure *P*_0_ reaches the material yield strength A, plastic deformation is initiated in the material.

(3) $$P_{0}=\sqrt[{3}]{\left[\left(\frac{6FE{*}^{2}}{\pi^{3}R^{2}}\right)\right]}=A$$

Solved:

(4) $$F=\left(\frac{A^{3}\pi^{3}R^{2}}{6E{*}^{2}}\right)$$

By substituting the parameters into Eq.(4), the critical static force required to initiate yielding is obtained as *F*≈11.15 N. However, this result is clearly unreasonable. The discrepancy arises from the simplifying assumption *P*_0_=A, which implies that yielding occurs when the maximum contact pressure at the surface center reaches the material yield strength. In reality, the stress state at the contact center is predominantly triaxial compression, under which yielding is unlikely to occur. Instead, yielding is more likely to initiate beneath the surface, where the maximum shear stress is developed. Therefore, the von Mises yield criterion provides a more accurate description of yielding behavior under multiaxial stress conditions.

Based on the von Mises yield criterion, yielding is evaluated using the equivalent stress as the governing parameter for plastic deformation. When applied to the Hertzian contact problem, it is found that the maximum von Mises stress does not occur at the contact surface, but rather at a subsurface location approximately 0.48a beneath the surface. The magnitude of this stress is typically lower than the maximum contact pressure at the center and can be approximated as:*σ_von Mises_*≈0.6*P_0_*,This implies that, in order for the equivalent stress at this critical subsurface location to reach the material yield strength, the actual contact pressure must be higher than that predicted by the classical Hertzian assumption. Therefore, a correction to the theoretical contact pressure is necessary when evaluating yielding under contact loading.When *σ_von Mises_ =* 1060 MPa, the corresponding equivalent contact pressure *P*_0_ can be obtained as Eq(5):Thus, the corrected maximum contact pressure required to initiate yielding is approximately 1767 MPa, which is significantly higher than the value predicted by the conventional Hertzian criterion.

(5) $$P_{0}=\frac{A}{0.6}=\frac{1060}{0.6}\approx 1767\;\text{MPa}$$

**Fig. 3:**
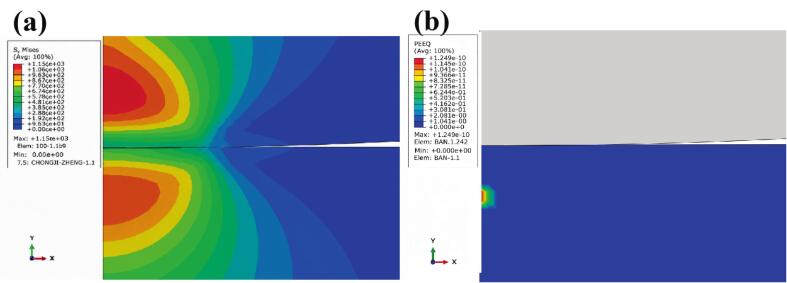
Finite element simulation results: (a) Mises contact stress at 48 N. (b) Simulated plastic strain at 48 N.

Recalculation indicates that the material enters the plastic regime only when the applied static load reaches approximately 48.5 N. By applying a static load of 48 N in the simulation, the von Mises contact stress distribution between the spherical indenter and the workpiece is obtained, as shown in Fig. 3(a), while the corresponding plastic strain field is presented in Fig. 3(b). The results show good agreement with the theoretical prediction of 48.5 N, both in terms of the critical load level and the location of plastic deformation initiation.

Based on this validation, the static load levels were selected as 20 N, 50 N, 100 N, and 500 N, corresponding to representative conditions where the material remains in the elastic regime, begins to yield, and experiences significant plastic deformation well beyond the yield limit, respectively.

### 2.2 The influence of static load and amplitude on RCS

**Fig. 4:**
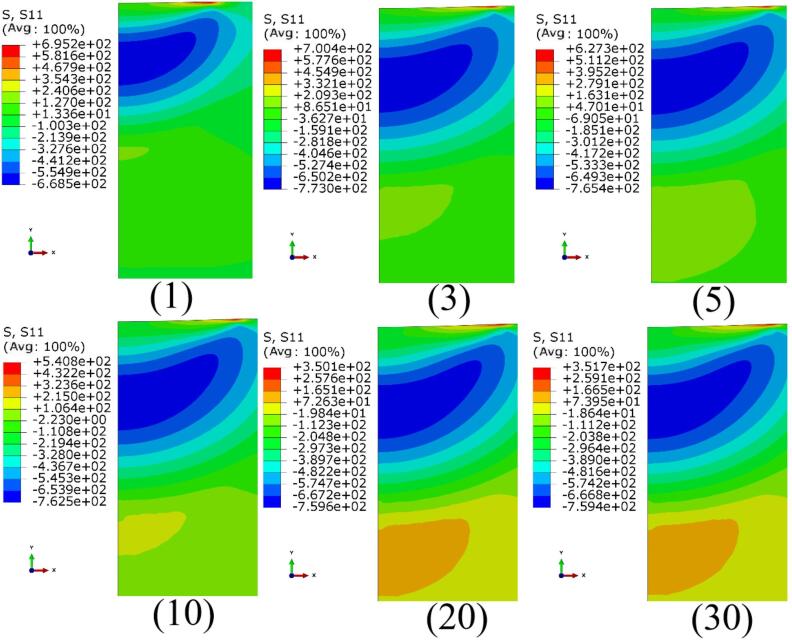
RCS contours for impacts from 1 to 30 times under a static load of 50 N and an amplitude of 10 μm.

**Fig. 5:**
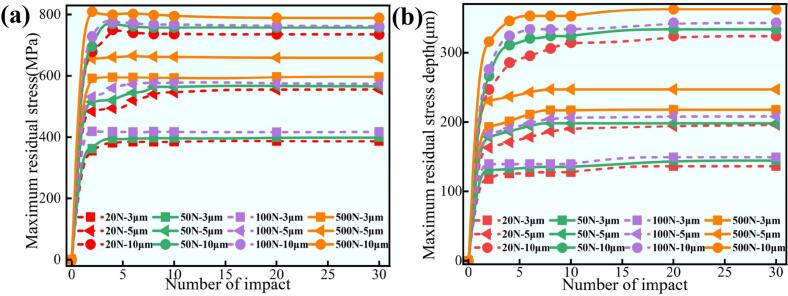
(a) Variation of maximum RCS with the number of impacts. (b) Variation of maximum RCS depth with the number of impacts.

As shown in Fig. 4, the RCS contours under a static load of 50 N and a vibration amplitude of 10 μm for different impact cycles indicate that the maximum RCS reaches its peak after the third impact. Subsequent impacts lead to a slight reduction in the RCS magnitude. Based on this observation, the evolution of the maximum RCS and its affected depth with respect to the number of impacts was extracted, and the corresponding trends are plotted in Fig. 5 for further analysis.

The simulation results in Fig. 5 reveal the influence of static load and vibration amplitude on both the magnitude and depth of RCS during the UIT. It is evident that the vibration amplitude plays a dominant role, significantly enhancing both the peak RCS and its penetration depth. While increasing either the static load or the amplitude contributes to strengthening the RCS field and extending its influence, the effect of static load remains relatively limited under the same amplitude condition. Only when the applied static load significantly exceeds the material yield strength does its influence become more pronounced.

As the vibration amplitude increases, the relative influence of static load gradually diminishes. Taking an amplitude of 3 μm as an example, the maximum RCS under static loads of 20 N and 500 N is 388.815 MPa and 597.021 MPa, respectively, with a difference of 208.206 MPa; the corresponding affected depths are 139.312 μm and 217.605 μm, differing by 78.239 μm. However, when the amplitude increases to 10 μm, the RCSes under 20 N and 500 N become 741.312 MPa and 788.753 MPa, respectively, with the difference reduced to 47.441 MPa; the corresponding depths are 323.656 μm and 362.225 μm, with a reduced difference of 38.594 μm. These results indicate that under high-amplitude conditions, the effect of static load on the magnitude of RCS becomes negligible, and its primary role shifts to promoting the deeper propagation of the RCS field.

During multiple impact processes, the evolution of maximum RCS and its depth exhibits both similarities and differences compared to the variation of impact depth and width. The similarity lies in that the depth of maximum RCS increases progressively with the number of impacts and approaches saturation after approximately six impacts, which is consistent with the trends of impact depth and width. In contrast, the maximum RCS itself reaches saturation as early as the third impact, with subsequent impacts having minimal influence. This discrepancy can be attributed to the formation mechanism of RCS, which originates from severe plastic deformation induced by impact and the constraint imposed by the elastic substrate during unloading. After the second impact, the incremental plastic deformation introduced by subsequent impacts is significantly smaller than that induced in the initial stages, and thus no longer leads to a substantial increase in RCS magnitude.

Based on the above simulation results, it can be preliminarily inferred that achieving an optimal RCS during the UIT does not necessarily require a high static load. Even under relatively low static load, a sufficiently high RCS level can be attained by appropriately increasing the vibration amplitude. Once the amplitude reaches a certain threshold, further increasing the static load has a negligible effect on enhancing the magnitude of RCS. Instead, its primary role is to enlarge the plastic deformation zone and promote the propagation of the RCS field into deeper regions.

Therefore, it can be concluded that static load mainly governs the impact morphology depth and the spatial distribution range of RCS, whereas the magnitude of RCS is predominantly controlled by the vibration amplitude.

### 2.3 Effect of coverage rate on RCS results

#### 2.3.1 Analysis of RCS evolution at the midpoint of the first impact under multiple impact superposition

**Fig. 6:**
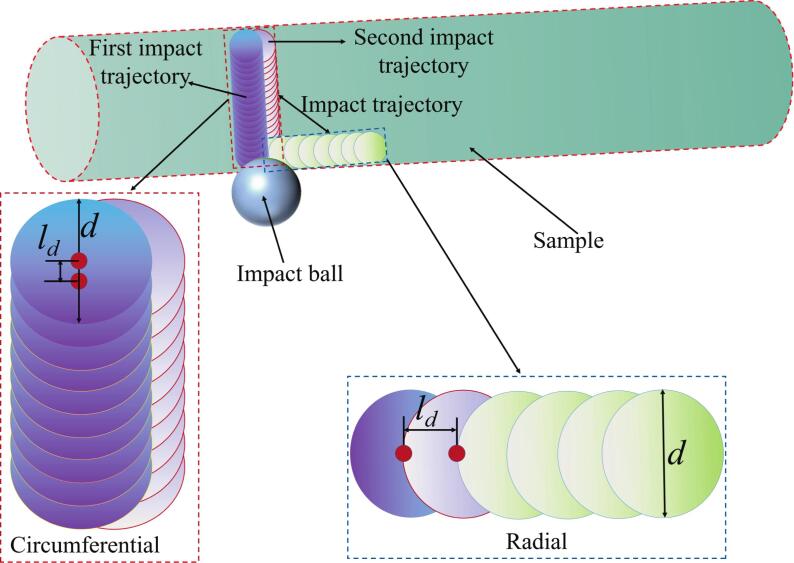
Schematic diagram of the definition of circumferential and axial coverage rates.

Although previous studies have revealed the fundamental effects of UIT on surface integrity, investigations into the cumulative effects of multiple impacts remain relatively limited. In this section, the influences of static load and vibration amplitude on the RCS field are systematically analyzed, with the aim of quantitatively characterizing the affected zone of UIT and its evolution with increasing impact cycles, thereby providing a basis for subsequent process parameter optimization. To further clarify the strengthening boundaries of UIT, parameter combinations of static loads of 20 N and 500 N, and amplitudes of 3 μm and 10 μm were selected. Simulations under different coverage conditions were then carried out to determine the upper and lower bounds of the strengthening effect. The schematic definition of coverage rate *η* is illustrated in Fig. 6, where *l_d_* represents the center-to-center distance between adjacent impact pits, and *d* denotes the diameter of a single impact pit.

(6) $$\eta =\frac{l_{d}}{d}$$

In UIT, the traverse speed is typically set within the range of 0.1～0.5 m/min. Taking the present conditions of a 27 kHz impact frequency and a traverse speed of 0.5 m/min as an example, the circumferential coverage rate can reach as high as 99.85%, which essentially corresponds to full coverage. Therefore, the influence of circumferential coverage is neglected in this study, and only the axial coverage is considered.

**Fig. 7:**
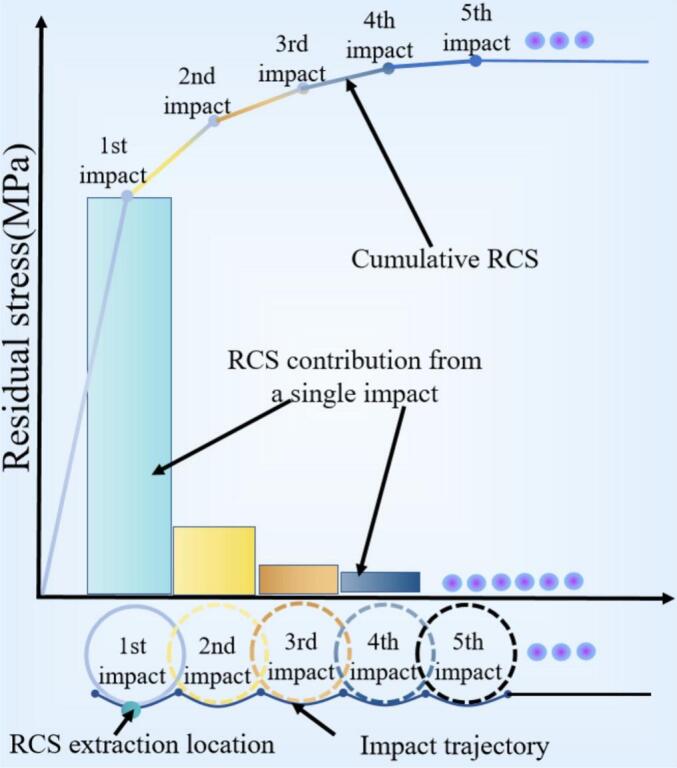
Schematic diagram of the region of the first impact affected by subsequent impacts.

During the UIT, the maximum RCS at the center of the first impact is affected by the superposition of subsequent impacts, and this influence gradually attenuates as the distance between impact points increases, as illustrated in Fig. 7. However, there is still a lack of studies quantitatively defining the effective range over which subsequent impacts influence the maximum RCS induced by the initial impact. To address this, the maximum RCS at the center of the first impact and its corresponding depth were first extracted, in order to investigate the spatial distribution of the effective influence zone during the UIT.

**Fig. 8:**
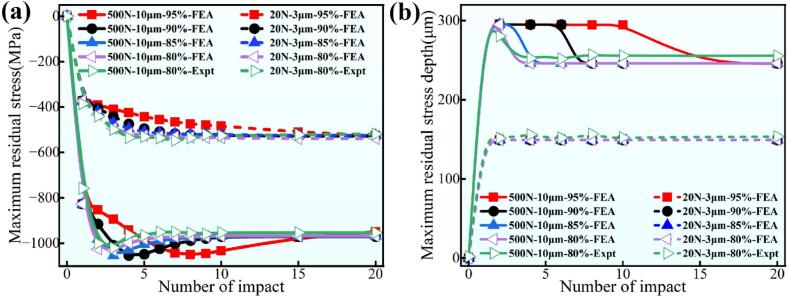
(a) Variation of RCS with the number of subsequent impacts. (b) Variation of RCS depth with the number of subsequent impacts.

Based on the variation trend of RCS with impact cycles shown in Fig. 7, the maximum RCS at the center of the first impact and its corresponding depth were extracted. Their evolution under subsequent impacts was analyzed, and the results are presented in Fig. 8.

As shown in Fig. 8, the influence of coverage rate on the final magnitude of RCS is relatively limited. The effective action range of a single impact is approximately comparable to the diameter of an individual impact pit. Taking a coverage rate of 90% as an example, regardless of the combination of static load and vibration amplitude, after ten impact cycles, subsequent impacts no longer produce a noticeable superposition effect on either the maximum RCS or its depth at the center of the first impact. These results provide a basis for defining the effective interaction range between adjacent impact points.

Furthermore, Fig. 8(b) indicates that the influence of subsequent impacts on the depth of maximum RCS at the first impact location is strongly dependent on the processing parameters. Under conditions of higher static load and larger amplitude, the depth of maximum RCS gradually shifts toward the surface with increasing impact cycles, whereas this phenomenon is not observed under lower parameter conditions. Based on these observations, it can be inferred that the effective influence range of UIT is approximately equivalent to the diameter (d) of a single impact pit. This finding provides useful guidance for parameter selection and for determining the effective superposition range of RCS in subsequent analyses.

#### 2.3.2 Multiple impact response variation of RCS at the midpoint of the impact trajectory

**Fig. 9:**
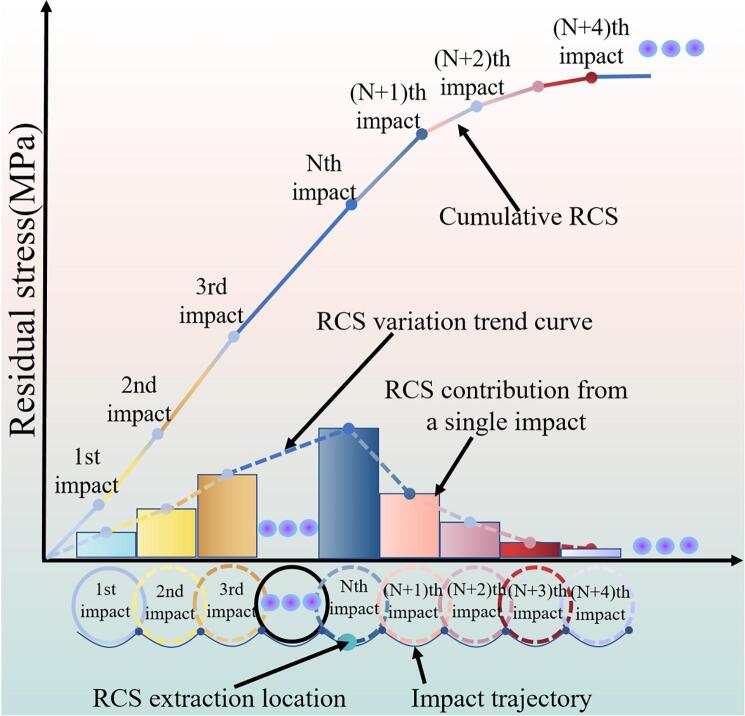
Schematic diagram of the region affected by multiple impacts at the midpoint of the Nth impact.

Based on the foregoing analysis, it is evident that the stress evolution at the first impact location during UIT is closely related to the positions of subsequent impacts, which provides a fundamental basis for analyzing the superposition behavior of RCS at a given point. The variation of RCS at the midpoint investigated in this section reflects the overall superposition characteristics under UIT as illustrated in Fig. 9.

To ensure a comprehensive evaluation, two additional representative parameter sets were introduced for the final analysis. Under conditions of low static load and low vibration amplitude, the maximum RCS at the midpoint remains essentially stable after reaching its peak, and subsequent impacts exhibit negligible regulatory effects. In contrast, under high-amplitude or high static load conditions, the RCS at the midpoint gradually decreases after reaching its maximum value. This behavior can be attributed to the long-range regulation effect of stress waves: the dynamic stress field generated by subsequent impacts superimposes on the pre-existing static RCS at the midpoint. When the combined stress exceeds the instantaneous yield strength of the work-hardened material, localized microplastic deformation is induced [35]. This mechanism, analogous to vibration-induced stress relaxation, releases part of the elastic strain energy initially “locked” at the midpoint, leading to a reduction in the peak RCS. As the impact location moves further away, the influence of stress waves diminishes and the RCS stabilizes, with the effective range consistent with previous findings, approximately equivalent to the width of a single impact pit.

**Fig. 10:**
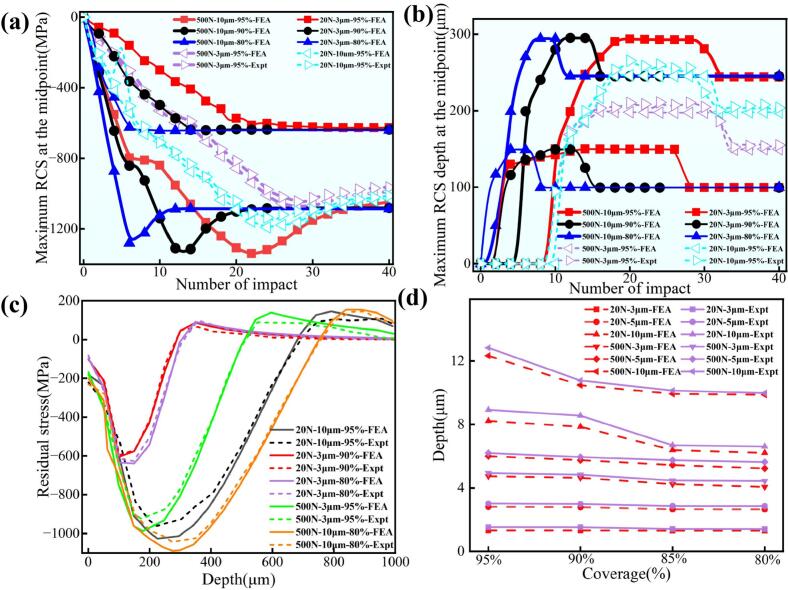
RCS and its depth results of materials after UIT. (a) RCS variation with impact number. (b) RCS depth variation with impact number. (c) RCS along the depth direction. (d) Impact depth variation with coverage rate.

As shown in Fig. 10, the stabilized RCS after UIT defines the upper and lower bounds of the strengthening effect, with a maximum of approximately 1081 ± 67 MPa and a minimum of about 625 MPa. At an amplitude of 10 μm, the final RCS under static loads of 20 N and 500 N are −1026.25 MPa and −1081.04 MPa, respectively, with a difference of only about 5%. Under low-amplitude conditions, a high static load can still achieve significant strengthening; for example, the 500 N–3 μm combination reaches −987.231 MPa after multiple impacts. This is because a higher static load establishes a broader stress gradient field, enabling even low-amplitude stress waves to superimpose effectively and induce plastic deformation. Conversely, although a lower static load produces a more limited stress field, sufficiently high-amplitude stress waves can still trigger deformation through superposition effects.Although the measured RCS magnitude approaches the initial uniaxial yield strength of 1Cr12Ni3Mo2VN steel, the two quantities should not be compared directly on a one-to-one basis. The X-ray measurement represents the in-plane residual stress component within the near-surface layer, whereas the yield strength reported in this study corresponds to the initial uniaxial yielding condition of the untreated bulk material. The severe plastic deformation and strain hardening induced by UIT can increase the local yield resistance of the strengthened surface layer. The repeatability of the X-ray measurements, together with the good agreement between the experimental and simulated stress-evolution trends, supports the reliability of the measured high-magnitude RCS.

Furthermore, Fig. 10(b) indicates that the depth of maximum RCS is nearly independent of coverage rate but evolves synchronously with the stress magnitude, reaching its maximum when the stress attains its peak. As shown in Fig. 10(d), both static load and vibration amplitude jointly influence the impact depth, and their combined effect leads to greater penetration depths. In addition, coverage rate plays a significant role: higher coverage results in increased depth, particularly under high-parameter conditions, suggesting that the coupling of static load and amplitude amplifies the effect of coverage on depth. Overall, both higher static load and larger vibration amplitude are beneficial for achieving a high-magnitude, deep RCS field.

(7) $$z\left(x,y\right)=\left\{\begin{array}{c} \begin{array}{cc} 300+110y+0.74x & y<5.2-0.0028x \end{array}\\ \begin{array}{cc} 520+60y+0.65x & 5.2-0.0028x<y< \end{array}\\ \begin{array}{cc} 980+0.2x & y>8.8-0.0046x \end{array} \end{array}\right)8.8-0.0046x$$

**Fig. 11:**
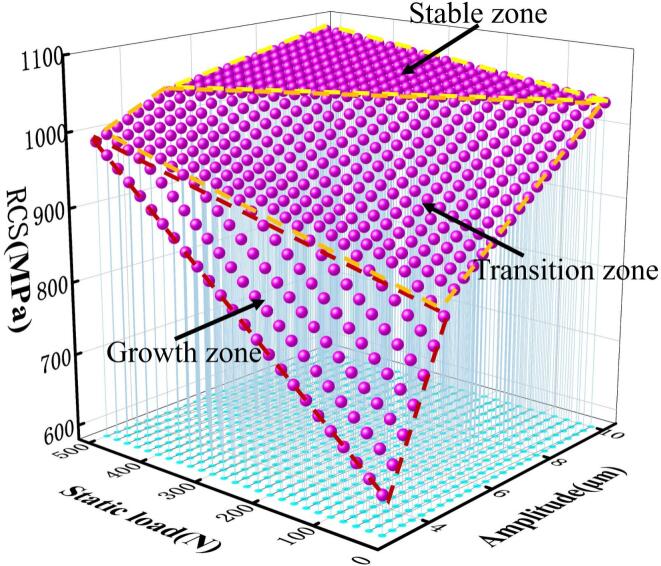
Fitted surface plot of RCS under different static load and amplitudes.

To clearly illustrate the effects of static load and vibration amplitude on RCS, a response surface was constructed, as shown in Fig. 11. The response surface can be divided into three distinct regions, with the boundary conditions described by Eq.(7), where *x* represents the static load and *y* denotes the vibration amplitude. Each region exhibits different response characteristics. In the growth region, the response is dominated by amplitude-dependent plastic deformation, resulting in a pronounced linear dependence on vibration amplitude. In the transition region, partial saturation of plastic deformation leads to a reduced slope with respect to amplitude. Finally, in the stable region, the response gradually stabilizes, and its dependence on both vibration amplitude and static load becomes weak, indicating that the strengthening effect induced by the impact parameters has reached a plateau.

## 3 Experimental verification

### 3.1 UIT and WE experiments

**Fig. 12:**
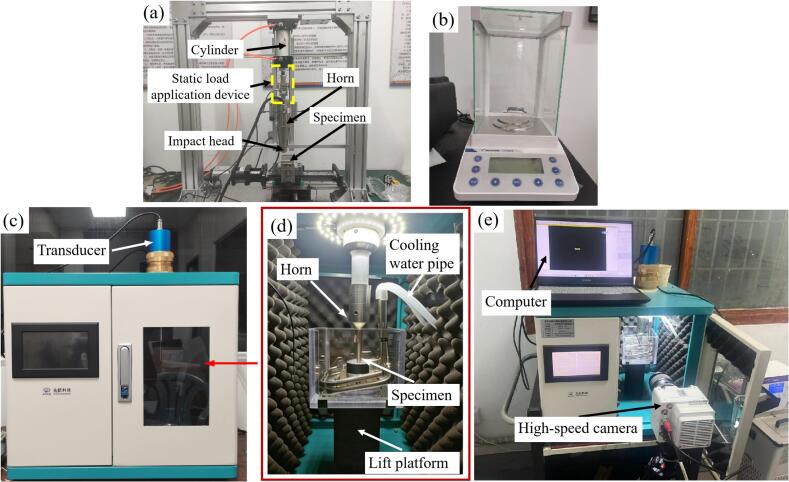
Experimental setup. (a) UIT. (b) FA224C electronic balance. (c) XOQS-2500 ultrasonic WE tester. (d) Internal placement diagram of the erosion tester. (e) Bubble capture image.

The UIT experiments were conducted using the equipment shown in Fig. 12(a), where the vibration amplitude was regulated via the control system, and the system resonance frequency *f* = 27 ± 0.8 kHz was an inherent characteristic of the device. A WC cemented carbide ball with a diameter of 14 mm was employed as the impact head. WE tests were carried out using an XOQS-2500 ultrasonic cavitation apparatus, as illustrated in Fig. 12(c–d), in accordance with international standard ASTM G32-16. The setup consists of an ultrasonic signal generator, a horn, and a temperature-controlled system, which induces the formation and collapse of cavitation bubbles on the specimen surface, thereby simulating erosion conditions experienced by steam turbine blades.

**Fig. 13:**
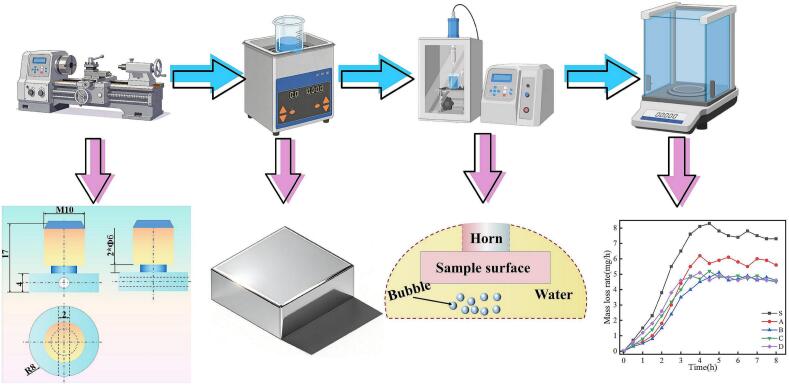
Experimental process.

During the experiments, the specimen was fixed at the tip of the horn, with deionized water used as the test medium. The liquid depth was maintained at 100 mm, and the specimen immersion depth was set to 10 mm. The solution temperature was controlled at 20 ± 2℃ using the thermostatic system. The ultrasonic vibration frequency was 20 kHz, with an amplitude of 50 μm and a working/interval cycle of 4 s/2 s. By characterizing the mass loss and surface damage morphology evolution during the WE process, the influence of UIT processing parameters on WE resistance was systematically evaluated. As shown in Fig. 12(e),To systematically evaluate the effects of UIT processing parameters on WE resistance, cavitation bubble behavior was recorded using a VEO 610 S high-speed camera manufactured by York Technology company., Ltd., USA. For each specimen, the frame exhibiting the maximum bubble number density within a single ultrasonic vibration cycle was selected as the representative image for quantitatively comparing the bubble density associated with different surface conditions. Fig. 13 illustrates the overall experimental procedure. First, turning sample preparation and ultrasonic impact treatment are performed, followed by cleaning and water erosion equivalent tests. Finally, mass loss is measured to evaluate the water erosion resistance under different parameters

### 3.2 Microstructure measurement

The WE tests were interrupted at eight selected time intervals (0 h, 0.5 h, 1 h, 1.5 h, 2 h, 4 h, 6 h, and 8 h) to perform intermediate measurements and characterizations. This approach aims to capture the initiation and rapid evolution of surface damage during the early stages of erosion, as well as the cumulative behavior during the steady-state stage. At each interval, the specimens were weighed using an FA224C electronic balance with an accuracy of 0.1 mg, as shown in Fig. 12(b), and the cumulative mass loss was recorded to obtain the erosion mass loss curves. Surface damage morphology was examined using a scanning electron microscope (Carl Zeiss Crossbeam 550), enabling detailed observation of the evolution of erosion pits, microcracks, and other damage features. Residual stress measurements were conducted using an X-ray diffraction system (Proto LXRD HDS-I, Cr-Kα radiation, sin^2^ψ method) to evaluate stress evolution during the erosion process. each processing condition, three independently prepared specimens were tested. Residual stress measurements were conducted at three representative locations within the treated region of each specimen to evaluate the uniformity of the stress distribution and the repeatability of the UIT process. By integrating these characterization techniques, the cumulative damage behavior and morphological evolution during WE were systematically obtained, providing a basis for in-depth analysis of the mechanisms by which UIT enhances erosion resistance.

## 4 Experimental results and analysis

### 4.1 Impact roughness results under different coverage rates

**Fig. 14:**
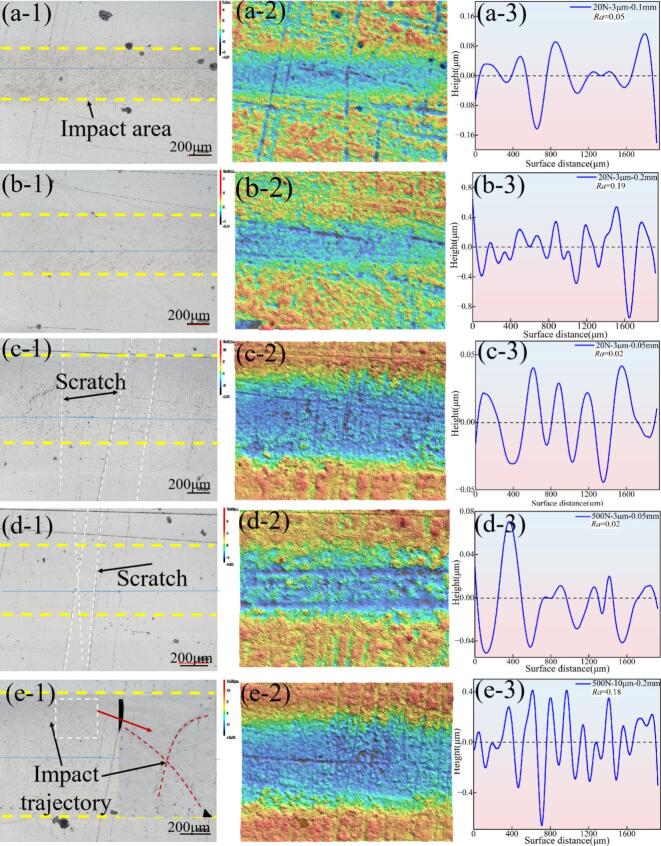
Experimental results of surface roughness under different impact parameters and offset distances.

Under low static load, the surface roughness in Fig. 14(a-3) is approximately *Ra*≈0.05 μm, whereas in Fig. 14(b-3) it increases to *Ra*≈0.19 μm. This difference is primarily attributed to the reduction in coverage rate. From a contact mechanics perspective, the Hertzian contact zones and the associated plastic deformation regions generated during impact do not sufficiently overlap, leading to non-uniform accumulation of plastic deformation. This results in pronounced surface height fluctuations and a significant increase in roughness. Therefore, coverage rate, by governing the spatial uniformity of impact energy distribution, becomes a key factor influencing surface roughness.

At higher amplitude with relatively low coverage, Fig. 14(c-3) shows that the roughness decreases to *Ra*≈0.02 μm, indicating effective surface smoothing. This suggests that increasing amplitude promotes sufficient plastic flow and surface reconstruction in the near-surface layer. High coverage ensures repeated overlapping impacts between adjacent craters, forming a continuous densified layer that suppresses residual asperities and reduces roughness. A further comparison with group (d) shows that *Ra*≈0.03 μm in Fig. 14(d-3), only slightly higher than that of group (c). This indicates that under conditions of sufficient coverage and high amplitude, the effect of static load on roughness exhibits a saturation behavior. While amplitude dominates the input of impact energy, static load mainly increases the initial contact stiffness and only slightly enlarges the plastic zone, thus having a limited influence on surface morphology.

In contrast, for group (e), the roughness increases significantly to *Ra* approx 0.18 μm, accompanied by pronounced surface undulations. This indicates surface deterioration under the combined effect of high static load and low coverage. The underlying mechanism is that excessive static load induces a highly constrained compressive stress state in the contact zone, leading to intensified localized lateral plastic flow and material pile-up, as evidenced by the impact traces shown in Fig. 14(e-1). Due to insufficient coverage, these plastically deformed regions are not subsequently “smoothed” by overlapping impacts, but instead accumulate along the impact path, ultimately resulting in roughness deterioration. Therefore, the key to roughness control lies not in simply increasing impact intensity, but in achieving a uniformly distributed plastic deformation field under high coverage conditions.

Combined with the previous finite element results, it is evident that achieving high-magnitude RCS does not necessarily require high static load. By properly matching vibration amplitude and coverage rate, it is possible to obtain both desirable RCS levels and improved surface quality, while avoiding surface roughening caused by excessive static load and localized material pile-up. Accordingly, the UIT experimental scheme listed in Table 2 was adopted in this study. Under moderate static load and high amplitude conditions, different offset distances were introduced to represent varying coverage rates. By comparing the results under different processing parameters, the roles of surface roughness and RCS in governing WE resistance were further elucidated.

**Table 2:** Experimental parameter.

Group	Static load/N	Amplitude/μm	Offset distance/mm
S	0	0	0
A	50	3	0.1
B	300	10	0.1
C	300	10	0.2
D	300	10	0.3

### 4.2 Microscopic structural properties

#### 4.2.1 Hardness result

**Fig. 15:**
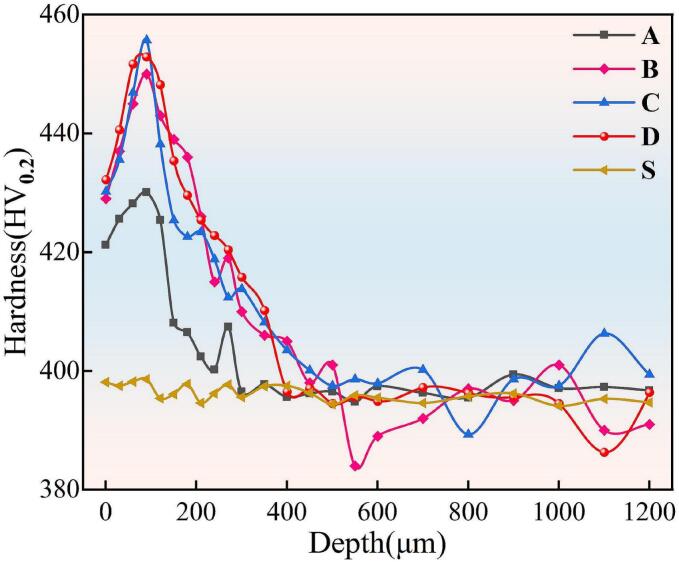
Hardness result**.**

Fig. 15 presents the cross-sectional microhardness profiles of the untreated and UIT-treated specimens. The hardness of the untreated specimen (S) remained nearly constant at 395–399 HV_0.2_ throughout the measured depth, indicating a relatively uniform initial mechanical state. In contrast, all UIT-treated specimens exhibited a pronounced hardness gradient. Specifically, the hardness gradually increased from the surface toward the subsurface region, reached a maximum value, and then progressively decreased to the substrate level with increasing depth.

The occurrence of the maximum hardness beneath the surface can be attributed to the subsurface distribution of impact-induced plastic strain. Under the repeated action of the spherical impact tip, the maximum equivalent stress and accumulated plastic deformation may develop below the contact surface. Consequently, the most pronounced work-hardened region is located within the near-surface subsurface layer rather than at the outermost surface. As the depth increases, both the impact-induced stress and plastic strain gradually attenuate, resulting in a progressive decrease in hardness.

Groups A and B had the same offset distance of 0.1 mm. When the static load was increased from 50 to 300 N and the vibration amplitude from 3 to 10 μm, the peak hardness increased from approximately 429 to 450 HV_0.2_, while the hardened region extended further into the substrate. This result indicates that an increase in impact energy promotes more intense and deeper plastic deformation. Groups B, C, and D were treated using the same static load and vibration amplitude, with only the offset distance being varied. These three groups exhibited comparable peak hardness values and similar attenuation trends along the depth direction, indicating that the offset distance had a limited effect on the through-thickness hardening response. The offset distance mainly controlled the lateral overlap between adjacent impact tracks and the uniformity of the treated surface, whereas the static load and vibration amplitude primarily determined the intensity and penetration depth of plastic deformation.

Therefore, the microhardness profiles confirm the formation of a gradient-hardened layer after UIT. Combined with the near-surface microstructural evolution revealed by EBSD, the results indicate that accumulated plastic strain, work hardening, and microstructural refinement jointly enhanced the resistance of the near-surface layer to localized deformation. This gradient-strengthened layer may contribute to delaying plastic strain accumulation and crack initiation under repeated cavitation impacts.

#### 4.2.2 EBSD result

**Fig. 16:**
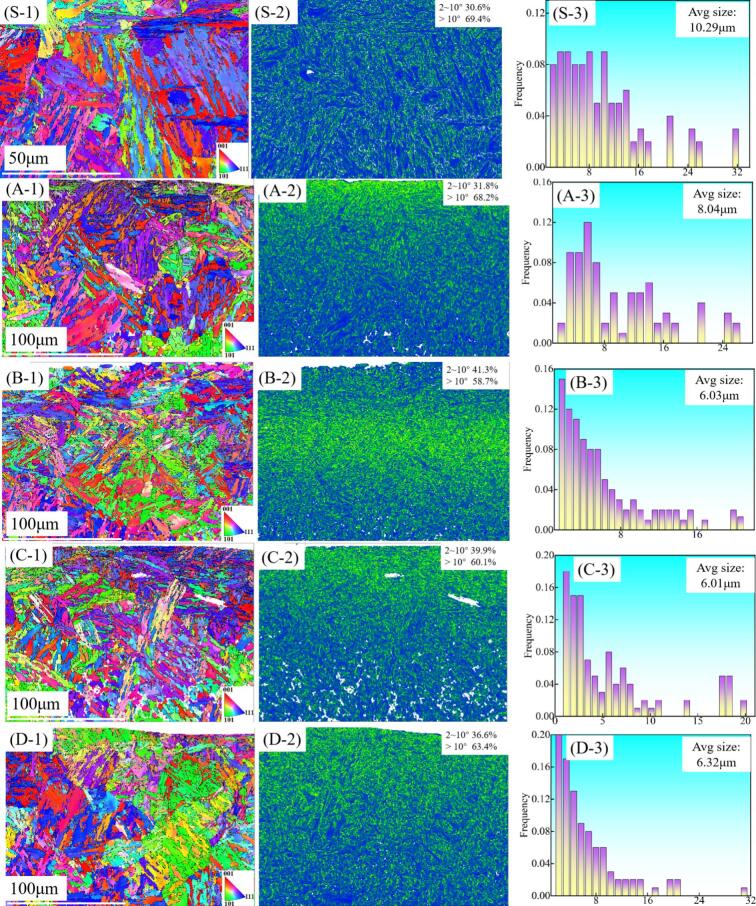
EBSD result. (1) IPF. (2) KAM. (3) Average grain size.

Fig. 16 presents the electron backscatter diffraction (EBSD) inverse pole figure (IPF) orientation maps, kernel average misorientation (KAM) distributions, grain-boundary misorientation statistics, and grain-size distributions of the untreated and UIT-treated specimens. The untreated specimen (Group S) exhibited a relatively coarse microstructure, with an average grain size of 10.29 μm. After UIT, the average grain sizes of Groups A, B, C, and D decreased to 8.04, 6.03, 6.01, and 6.32 μm, respectively, indicating that repeated ultrasonic impacts induced pronounced grain refinement in the near-surface region.

The degree of grain refinement was closely related to the impact energy. Compared with Group A, Groups B–D, which were treated under higher static load and vibration amplitudes, exhibited markedly smaller average grain sizes of approximately 6 μm. This result indicates that increasing the static load and vibration amplitude intensifies near-surface plastic deformation and accelerates the evolution of coarse grains into refined grains. In contrast, the grain sizes of Groups B, C, and D were relatively similar, suggesting that, within the parameter range investigated in this study, variations in impact coverage had a smaller effect on the final characteristic grain size than the static load and vibration amplitude.

The KAM distributions and grain-boundary statistics further reveal the deformation characteristics induced by UIT. The fraction of low-angle grain boundaries with misorientation angles of 2°–10° was 30.6% in Group S and increased to 31.8%, 41.3%, 39.9%, and 36.6% in Groups A, B, C, and D, respectively. The increased local misorientation and low-angle grain-boundary fractions in the UIT-treated specimens indicate more pronounced lattice rotation, dislocation accumulation, and subgrain-boundary formation during repeated impact loading.Among the UIT-treated specimens, Group B exhibited the highest fraction of low-angle grain boundaries, followed by Groups C and D. Because Groups B–D were treated using the same static load and vibration amplitude and differed only in impact offset distance, this trend suggests that impact coverage primarily affects the accumulation and spatial uniformity of plastic deformation. A smaller impact offset increases the overlap between adjacent impact tracks, enabling repeated plastic deformation to accumulate more continuously and thereby producing a more pronounced local orientation gradient. When the offset distance was increased to that used for Group D, the overlap between adjacent impact tracks decreased and the accumulation of plastic deformation was weakened, although pronounced grain refinement remained evident.

The microstructural evolution revealed by EBSD can improve WE resistance through several mechanisms. First, grain refinement increases the number of grain boundaries, which impede dislocation motion and thereby enhance the resistance of the near-surface material to localized plastic deformation. Second, the increased local misorientation and low-angle grain-boundary fraction reflect dislocation accumulation and subgrain formation. These features may improve strain-hardening capacity and promote a more uniform distribution of plastic strain under repeated microjet and water-hammer loading. Consequently, severe strain localization at isolated surface defects can be mitigated, delaying the initiation of cavitation-induced microcracks.

In addition, the refined grain structure and increased grain-boundary density can deflect and lengthen crack-propagation paths, thereby increasing the energy required for continued crack growth and material detachment. This interpretation is consistent with the microhardness–depth profiles, which show that all UIT-treated specimens developed a pronounced gradient-hardened layer. Therefore, the combined effects of grain refinement, dislocation-related lattice distortion, subgrain-boundary formation, and gradient hardening enhance the resistance of the near-surface region to cyclic plastic deformation and crack propagation.

### 4.3 Bubble nucleation and experimental observations

#### 4.3.1 Contact angle and surface energy analysis

**Fig. 17:**
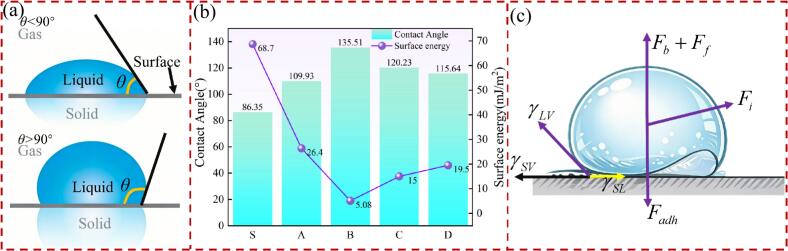
(a) Schematic diagram of contact angle. (b) Measurement results of contact angle and surface energy. (c) Forces acting on a bubble.

The WE resistance of a material surface is closely related to its surface free energy, which can be quantitatively characterized by contact angle measurements. To further elucidate the influence of UIT on the interfacial properties of 1Cr12NiMo2VN steel, systematic contact angle measurements were conducted. As shown in Fig. 17, the untreated substrate exhibits a contact angle of 86.35°, indicating a weakly hydrophilic surface.

After UIT, the contact angle shows a significant increasing trend, with a clear dependence on coverage rate. Under high coverage conditions (Groups A and B), the contact angles increase to 109.93° and 135.51°, representing improvements of approximately 27.2% and 56.9%, respectively, compared to the initial state. This indicates a transition of the material surface from hydrophilic to distinctly hydrophobic. As the coverage rate decreases (Groups C and D), the contact angles reduce to 120.23° and 115.64°, respectively. Although lower than the peak values, they remain within the hydrophobic regime, suggesting that the enhancement in surface hydrophobicity induced by UIT is relatively stable. Overall, these results demonstrate that higher coverage rates are more favorable for establishing a stable hydrophobic interfacial state.

According to interfacial thermodynamics, variations in the contact angle reflect the redistribution of interfacial energies among the solid–liquid–gas phases. The surface free energy of the solid can be described based on the Owens–Wendt–Rabel–Kaelble (OWRK) model, as expressed in Eq.(8) [36], [37]:

(8) $$\gamma_{L}\left(1+\cos\theta \right)=2\left(\sqrt{\gamma_{S}^{D}\gamma_{L}^{D}}+\sqrt{\gamma_{S}^{P}\gamma_{L}^{P}}\right)$$

Based on the parameter values reported by Li [36], the surface free energy was calculated from the measured contact angles. The results show a pronounced decreasing trend in surface free energy with increasing contact angle: it drops significantly from 68.7 mJ*m^−2^ in the initial state to 26.4 mJ*m^−2^ after UIT, and further decreases to 5.08 mJ*m^−2^ with increased static load, corresponding to a reduction of up to 92.6%. As the coverage rate decreases, the surface free energy rises to 15 mJ*m^−2^ and 19.5 mJ*m^−2^, respectively, but remains substantially lower than that of the untreated surface. These results indicate that high coverage conditions promote the transition of the material surface from a high-energy state to a low-energy state.

(9) $$V=\frac{K(F_{\mathrm{ad}}-190)}{3\lambda }∙{\left(\frac{b}{a}\right)}^{3}∙\left(\frac{P∙L}{P_{m}}\right)$$

From the perspective of classical heterogeneous nucleation theory [38], an increase in contact angle and the corresponding decrease in solid surface energy significantly raise the Gibbs free energy barrier for cavitation inception Δ*G*, which can be described by Eq.(10). The geometric factor *f*(*θ*) increases monotonically with contact angle. Therefore, increasing the contact angle effectively elevates *f*(*θ*), thereby raising the energy barrier for bubble nucleation at the solid–liquid interface. As a result, a higher negative pressure is required for bubble formation and growth.

Within the framework of the Harvey crevice model, reverse pressure within surface microcavities can stably confine gas nuclei inside the crevices, preventing their expansion under negative pressure conditions. Consequently, the increase in contact angle and reduction in surface energy not only delay heterogeneous nucleation but also reduce the number of generated bubbles and suppress their subsequent growth.

(10) $$\Delta G^{*}=\Delta Gf\left(\theta \right)=\frac{\Delta G\left(2+\cos\theta \right){\left(1-\cos\theta \right)}^{\frac{1}{2}}}{4}$$

To further elucidate the intrinsic mechanism by which UIT enhances the WE resistance of the material, the regulation of bubble behavior by surface energy is analyzed from the perspective of bubble dynamics. The attachment and detachment of bubbles on a solid surface are governed by the interfacial energy balance among the solid–liquid–gas phases. When a bubble detaches from the surface, the force balance can be expressed by Eq.(11), where *F_i_* denotes the inertial force, *F_b_* represents the buoyancy force, and *F_f_* corresponds to the hydrodynamic shear force.

(11) $$F_{i}+F_{b}+F_{f}>F_{\mathrm{adh}}$$

By combining the classical thermodynamic Dupré equation Eq.(12) with the Young equation Eq.(13) [39], [40], the surface adhesion force can be expressed as follows:

(12) $$W_{a}=\gamma_{\mathrm{SV}}+\gamma_{\mathrm{LV}}-\gamma_{\mathrm{SL}}$$

(13) $$\gamma_{\mathrm{SV}}-\gamma_{\mathrm{SL}}=\gamma_{\mathrm{LV}}\text{(1}+\cos\left(\theta \right))$$

(14) $$F_{\mathrm{adh}}=k\left(r\right)\gamma_{\mathrm{LV}}\text{(1}+\cos(\theta \text{))}$$

When the solid surface energy γ*_SV_* decreases, the solid–liquid interfacial tension *γ_SL_* correspondingly increases, leading to an increase in contact angle and a transition of the surface from hydrophilic to hydrophobic. According to the above relationships, the reduction in surface energy results in a significant decrease in the adhesion force *F_adh_*, which is consistent with the findings reported by Sun [39]. The direct mechanical consequence of this interfacial effect is that the work of adhesion between wall-adhered cavitation bubbles and the solid substrate is substantially reduced, and the pinning effect at the three-phase contact line is greatly weakened. As a result, bubbles can overcome the surface retention force induced by contact angle hysteresis under relatively fluid drag or buoyancy, leading to sliding detachment at sizes much smaller than the critical growth radius.Therefore, the reduction in surface energy not only increases the energy barrier for heterogeneous nucleation from a thermodynamic perspective, thereby suppressing cavitation inception, but also promotes the premature detachment of bubbles from the surface by weakening solid–liquid adhesion from an interfacial dynamics standpoint. This constitutes a dual mechanism by which surface engineering effectively regulates cavitation-induced damage.

#### 4.3.2 Observation of cavitation bubbles

**Fig. 18:**
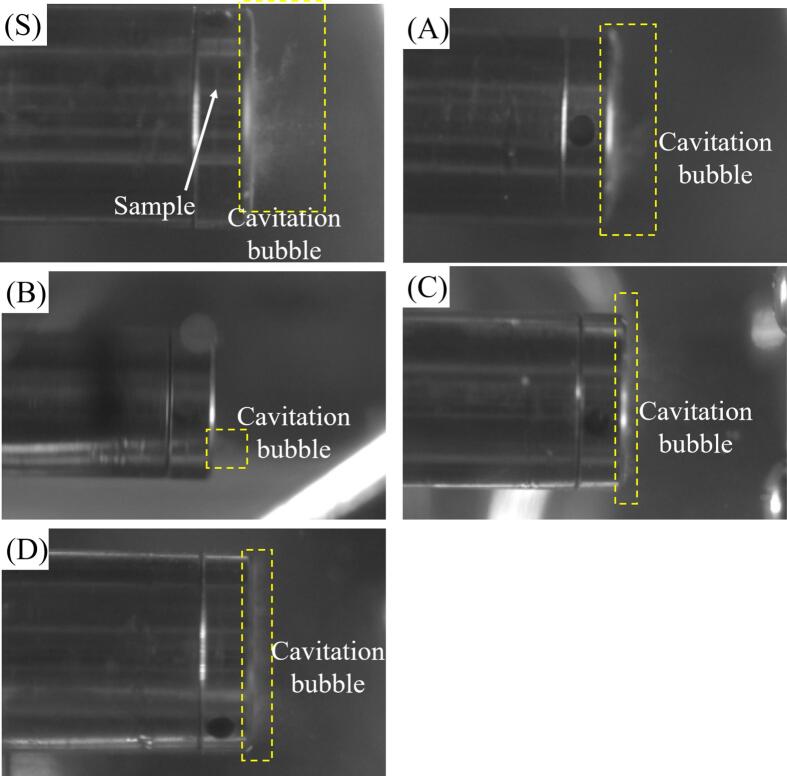
Observation of bubbles during the initial stage of water erosion.

Fig. 18 presents the initial near-surface bubble distributions of the untreated and UIT-treated specimens under identical cavitation conditions. During the initial stage of cavitation, the untreated specimen, which exhibited the highest surface roughness, developed a relatively continuous bubble-cloud structure over a comparatively large area near the surface. This observation suggests pronounced bubble accumulation and bubble–bubble interactions in the vicinity of the solid–liquid interface. In contrast, the improved surface finish of all UIT-treated specimens resulted in more localized and discrete bubble distributions, accompanied by reduced continuity of the bubble clouds.

Differences in bubble distribution among the UIT-treated groups were also associated with the processing parameters. Group A exhibited a relatively high visible bubble population, whereas Groups B and C showed more discrete bubble distributions after treatment at higher impact energies. Group D, which had a lower surface roughness than Groups B and C, exhibited a less spatially uniform and more localized bubble distribution. Qualitative analysis of the selected observation regions showed that the overall bubble number density of the UIT-treated specimens was lower than that of the untreated specimen, indicating a reduction in the level of near-surface cavitation activity. This observation is consistent with the reduction in surface roughness induced by UIT. A smoother surface contains fewer asperities, micropits, and other potential gas-trapping sites, thereby reducing the number of favorable locations for gas retention and heterogeneous bubble attachment. In addition, the increased contact angle and reduced surface energy may affect bubble attachment and detachment behavior at the solid–liquid interface.

In the present study, these observations are used as visual and semi-quantitative evidence of differences in near-surface cavitation activity. The observed trend is consistent with the reduction in surface roughness and the improvement in WE resistance. Nevertheless, the bubble number density derived from optical images should not be regarded as a direct measure of cavitation intensity, because cavitation intensity is also governed by multiple factors, including the acoustic-pressure amplitude, bubble-collapse dynamics, dissolved-gas content, and local flow-field conditions.

In addition, the bubble distributions during the later stage of WE were further examined. The results showed that, as the specimen surfaces were progressively damaged under prolonged cavitation exposure, the bubble number densities and spatial distributions of the different specimens gradually became similar. This observation suggests that the influence of the initial surface roughness on cavitation behavior diminished during the later erosion stage.

Under these conditions, the dominant mechanism gradually transitioned from roughness-controlled bubble attachment to a subsurface-stress-dominated damage-resistance mechanism. Although the surface conditions of the specimens became increasingly similar during the later stage of WE, the UIT-treated specimens continued to exhibit superior WE resistance, indicating that the RCS field and the gradient-strengthened subsurface layer became increasingly important. In particular, the subsurface RCS field reduced the effective transient tensile stress induced by cavitation bubble collapse, thereby suppressing crack initiation and delaying material detachment.

**Fig. 19:**
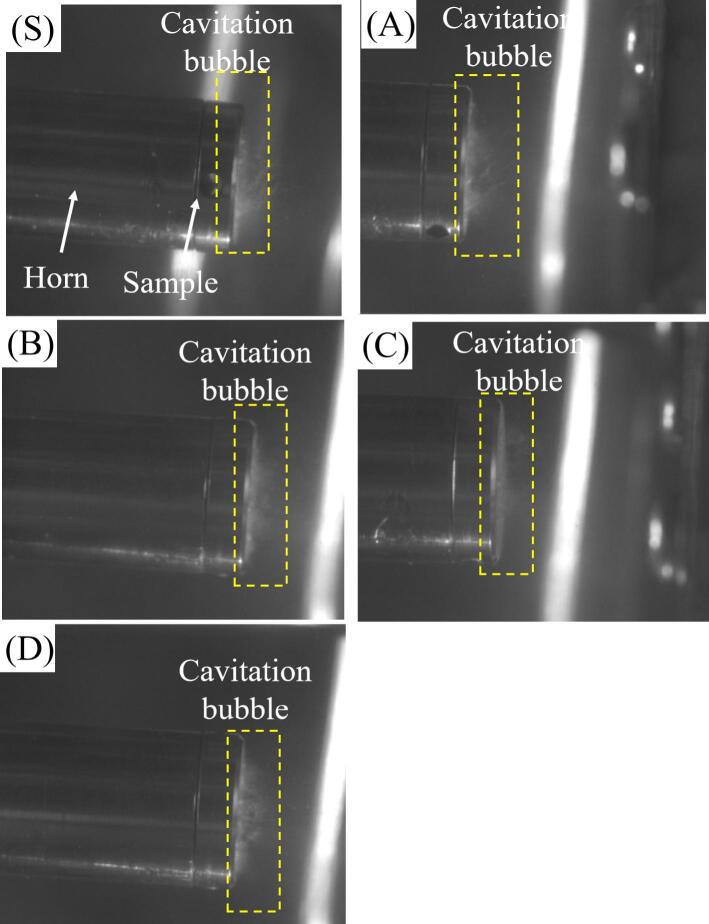
Observation of bubbles during the late stage of WE.

Fig. 19 presents the bubble distributions of the different specimens during the later stage of WE. It can be observed that the bubble distributions over specimens S, A, B, C, and D became increasingly similar at this stage. The near-surface bubble clouds exhibited more diffuse and spatially uniform characteristics, while the differences in bubble number density among the specimens were markedly reduced. These observations indicate that the influence of surface roughness on cavitation behavior gradually diminished as WE progressed. The surface topographies introduced or modified by UIT were progressively altered under continuous cavitation exposure, causing the surface conditions of the specimens to converge and reducing the sensitivity of bubble attachment and cavitation distribution to the initial surface-state differences.

Under these conditions, the dominant cavitation-response mechanism gradually shifted from surface-topography control to subsurface mechanical-property control. Although the differences in surface roughness were substantially reduced during the later stage, the UIT-treated specimens still exhibited lower mass loss and superior WE resistance. This result indicates that the RCS field and the associated gradient-hardened layer became the primary damage-resistance factors at this stage. RCS reduced the effective transient tensile stress induced by cavitation bubble collapse, thereby suppressing crack initiation and delaying material detachment. Therefore, the bubble observations during the later stage support a transition in the dominant mechanism from “roughness-controlled bubble behavior” in the early stage to “residual-stress- and microstructure-controlled damage resistance” in the later stage.

### 4.4 Cumulative quality loss analysis

**Fig. 20:**
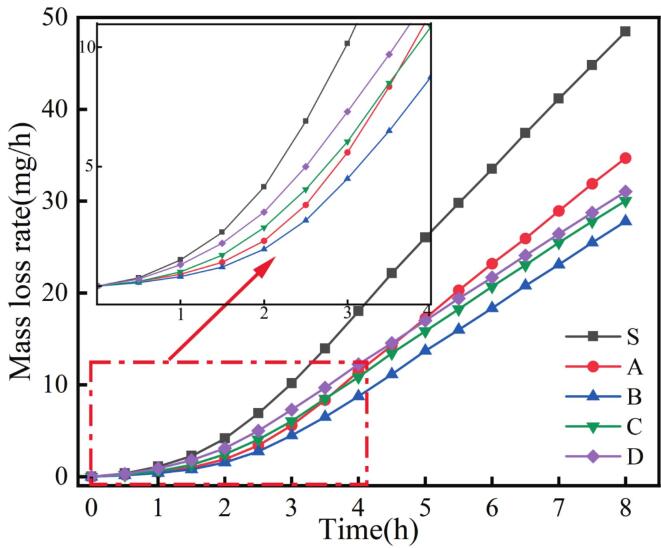
Cumulative mass loss curves of specimens under different process conditions.

Fig. 20 presents the cumulative mass loss curves of the untreated specimen and four groups of UIT 1Cr12Ni3Mo2VN stainless steel specimens after 8 h of WE in deionized water. The WE resistance *q*(*w,h,t*) can be defined as [8],as expressed in Eq.(15). In this study, the WE resistance is indirectly evaluated through the measured mass loss, where a lower cumulative mass loss corresponds to superior WE resistance.

(15) $$q\left(w,h,t\right)=\frac{1}{\mathrm{dV}}\sum_{i=1}^{J}\frac{4\pi }{3}R_{i}^{3}\left(t\right)$$

During the initial stage of WE, no significant mass loss is observed for all five groups; however, the untreated specimen exhibits slightly higher mass loss compared to those subjected to UIT. As the erosion time increases, the cumulative mass loss of all specimens shows a linear growth trend during the acceleration stage. After 8 h of effective cavitation erosion, the cumulative mass loss of the untreated specimen reaches 47.3 mg. In comparison, the treated specimens exhibit reductions in mass loss of 26.64%, 41.23%, 36.47%, and 34.36%, respectively.

Based on the cumulative mass loss, the average erosion depth of the specimen surface can be calculated. The corresponding expression is given in Eq.(16) as follows:

(16) $$H=\frac{\Delta M}{\rho S}$$

**Fig. 21:**
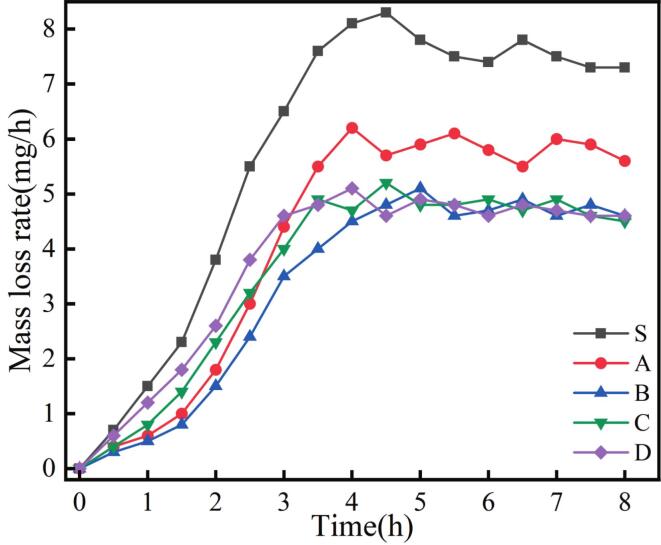
Mass loss rate curves of specimens under different process conditions.

Fig. 21 compares the cumulative mass loss curves of the untreated specimen and those treated under different UIT parameters. The incubation period of the untreated specimen lasts only about 0.5 h, whereas for the treated specimens it is extended to approximately 1.5 h. This indicates that the duration of the incubation stage is primarily governed by surface roughness: lower roughness leads to a longer incubation period, which is consistent with the findings of Kirols [14], here excessive roughness accelerates damage initiation.

Upon entering the acceleration stage, the initial mass loss rate remains strongly dependent on surface roughness, with smoother specimens exhibiting slower material loss. In the mid-to-late acceleration stage, although surface damage becomes evident in the three groups treated under high static load and high amplitude, their mass loss rates tend to converge. In contrast, the A-group specimen shows a progressively increasing loss rate, exceeding that of the other modified groups by 3 h. The acceleration stage ends at approximately 3.5 h for the untreated specimen and 4.5 h for the treated specimens, after which the process transitions into the deceleration stage.The treated specimens exhibit a significantly prolonged deceleration stage, during which the mass loss rate decreases gradually. This behavior is closely associated with the subsurface RCS introduced by UIT. All treated specimens enter a steady-state stage after approximately 6 h. Notably, the onset of the steady-state stage is not determined by whether the erosion depth exceeds the depth of peak RCS, but rather by a dynamic equilibrium governed by the gradient distribution of the RCS field. As erosion progresses into regions where RCS increases, the inhibitory effect of compressive stress on crack propagation becomes more pronounced, eventually balancing the driving force of damage, which manifests as a nearly constant mass loss rate.

### Analysis of the morphology of WE damage

**Fig. 22:**
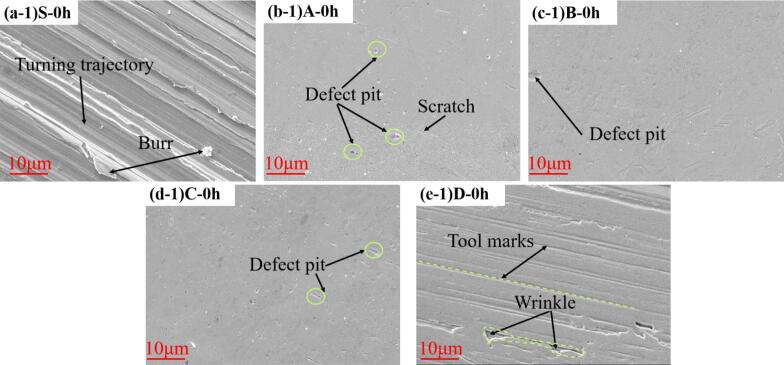
Surface damage morphology under different processes.

Fig. 22 systematically illustrates the evolution of surface morphology for specimens treated under different UIT parameters during the WE process. Prior to erosion (a1 ∼ e1), the surface roughness of all treated specimens is significantly reduced compared to the untreated state, and the degree of improvement is closely related to the impact spacing. When the spacing is ≤ 0.2 mm, substantial overlap of impact tracks occurs, effectively eliminating surface asperities and geometric discontinuities. In contrast, excessive spacing leads to insufficient coverage, leaving untreated regions between adjacent impact craters, thereby weakening the roughness improvement. These unfavorable microstructural features provide preferential sites for cavitation bubble nucleation and attachment.

**Fig. 23:**
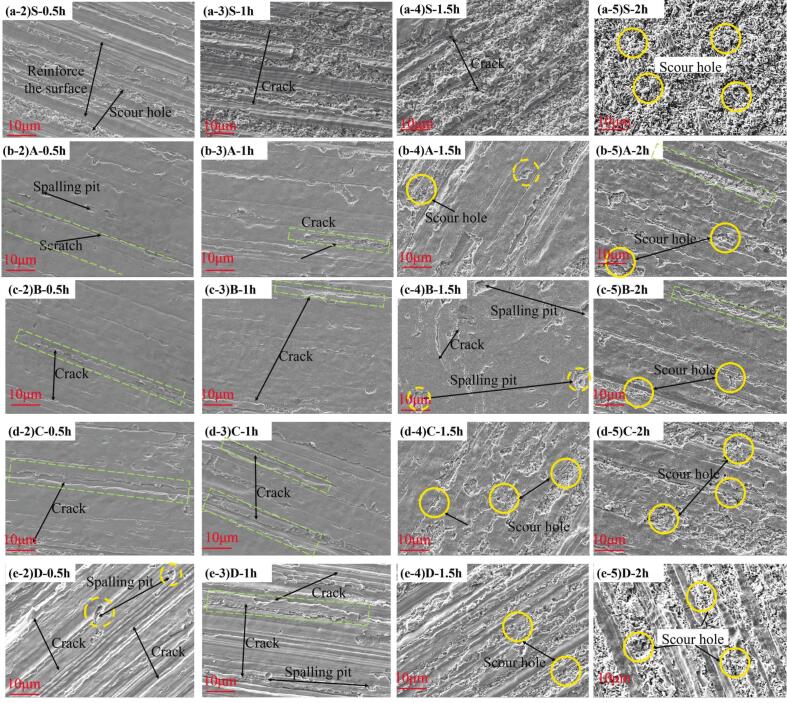
Surface damage morphology of specimens subjected to effective WE for 0–2 h under different processes. (All markings in the figure indicate favorable factors for bubble nucleation.).

As shown in Fig. 23, during the early stage of WE, the untreated specimen surface is rapidly covered by a high density of erosion pits and cracks, indicating severe local material removal at the surface. In contrast, the ultrasonically impacted specimens exhibit markedly improved resistance: for Groups A, B, and C, large portions of the original surface morphology are still preserved, demonstrating enhanced resistance to cavitation-induced damage.

During the 0.5–1 h interval, the untreated (S group) specimen, due to its relatively high initial roughness, facilitates bubble nucleation within surface depressions. These bubbles grow and collapse, generating high-amplitude transient microjets that act on surface asperities leading to localized microcracking and material spallation [41]. By comparison, the treated specimens maintain higher surface integrity, with only slight localized material removal observed, and the degree of damage increases with increasing impact spacing. This behavior arises because the smoother surface produced by UIT a higher Gibbs free energy barrier for nucleation, effectively suppressing cavitation bubble formation and thereby prolonging the incubation period.

In conjunction with classical cavitation nucleation theory [42], [43], surface micro-crevices are recognized as primary sites for bubble nucleation, and their geometric characteristics directly govern nucleation behavior. Considering a conical cavity with a half-angle α and an opening radius *r_0_*, the critical radius *R_c_* of a stable gas nucleus (assumed spherical cap-shaped) can be described by Eq.(17). As UIT reduces surface roughness, previously narrow and sharp cavities become smoother and more open, leading to an increase in α According to the critical curvature relationship, an increase in α results in a larger critical nucleation radius *R_c_*, thereby making bubble formation more difficult. When α > 90°, the pressure conditions promote rapid gas escape, preventing the formation of stable nuclei [44], thus, such cases fall outside the scope of the crevice-based nucleation model.This relationship mechanistically explains how reduced surface roughness suppresses bubble nucleation. In this context, *P_v_* represents the saturated vapor pressure of the liquid, and *P_L_* denotes the internal liquid pressure.

(17) $$R_{c}=\frac{2\sigma }{P_{v}-P_{l}}\frac{1}{\cos\alpha }$$

As WE progresses to 1.5 h and 2 h, the surface layer of the untreated specimen is almost completely removed, resulting in a high density of erosion pits. These local defects act as preferential sites for bubble nucleation and growth, thereby accelerating material removal. In contrast, the UIT specimens exhibit a significantly delayed damage evolution. For Groups A and B, initial surface spallation begins to appear after 1.5 h, and the affected area expands markedly by 2 h. Although Group D performs better than the untreated specimen, its insufficient impact coverage leads to limited improvement in surface roughness, and thus a higher degree of damage compared to the other treated groups.

These results indicate that once the erosion process enters the development stage, the micro-pits formed in the early stage become new nucleation sites, further accelerating WE damage. In regions with lower roughness, defects exhibit smaller stress concentration factors, which effectively mitigate the transient high-stress pulses generated during bubble collapse. As a result, the initiation of microcracks is delayed, significantly prolonging the retention of surface integrity.

**Fig. 24:**
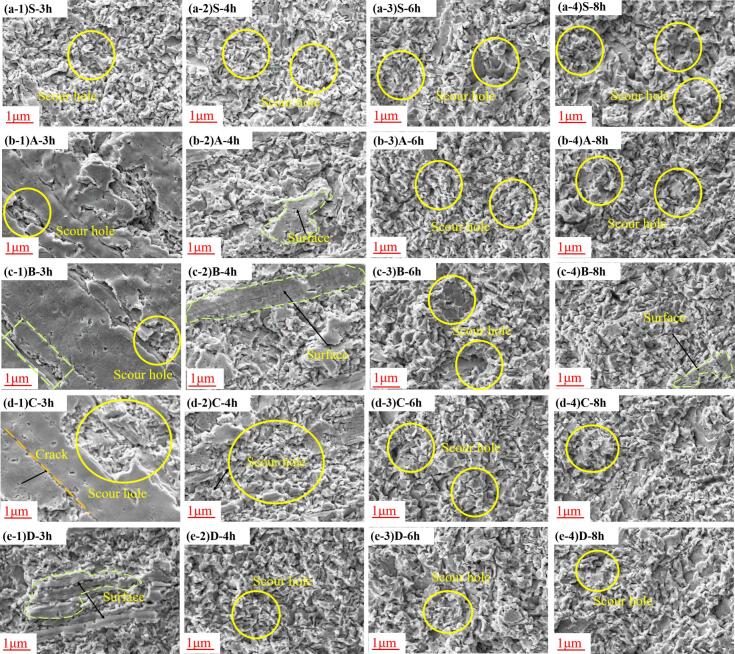
Surface damage morphology of specimens subjected to effective WE for 3–8 h under different process conditions.

Fig. 24 illustrates the evolution of surface erosion morphologies for different specimens after 3 h of WE. When the erosion time is extended to 4 h, all specimens exhibit high-density erosion pits and a network of semi-penetrating cracks, indicating that the surface layer has been almost completely removed. At this stage, the damage mechanism transitions from surface-dominated behavior to subsurface-controlled response, and the RCS field begins to govern the subsequent damage evolution.

In conjunction with the mass loss results in Fig. 20, the untreated specimen, lacking an effective RCS barrier, shows rapid crack propagation under cyclic impact loading and thus exhibits the highest steady-state mass loss rate. For Group A, the relatively low level of induced residual compressive stress results in insufficient WE resistance, leading to a higher mass loss rate compared to the other three groups with higher residual stresses. In contrast, the three high-residual-stress groups exhibit similar mass loss rates, suggesting that once the residual compressive stress exceeds a certain threshold, its contribution to cavitation resistance becomes comparable. This strengthening mechanism can be further interpreted using the fatigue-based erosion model proposed by Slot [45]:

The RCS field acts as a barrier that external tensile stresses must overcome to drive crack propagation, thereby significantly increasing the crack growth threshold. Moreover, the transient tensile stresses generated during bubble collapse are superimposed with the RCS, effectively reducing the net tensile stress experienced by the material and consequently slowing down the fatigue crack propagation rate.

In summary, the essence of improving WE resistance lies in a synergistic mechanism. On one hand, reducing surface roughness significantly increases the Gibbs free energy barrier for bubble nucleation, effectively blocking preferential invasion paths along surface defects and thereby enhancing resistance during the early stage of erosion. On the other hand, high-magnitude RCS establishes an internal mechanical barrier against stress-induced damage, enabling the material to maintain superior cavitation resistance even in the steady-state stage.

### 4.6 Mechanism analysis

The following theoretical analysis is intended to provide an order-of-magnitude interpretation of the mechanical loading generated by cavitation bubble collapse, rather than to precisely reconstruct the transient cavitation field in the present experiments. The analysis is based on the following simplifying assumptions:

(1) A representative isolated bubble is considered, and the bubble is assumed to remain approximately spherical before collapse;

(2) The bubble is assumed to collapse near a locally flat solid surface, producing an axisymmetric microjet normal to the material surface;

(3) The liquid properties are assumed to remain constant, while the effects of temperature variation, dissolved gas, liquid viscosity, and thermal dissipation are neglected;

(4) Bubble–bubble interactions, bubble-cloud effects, microjet deflection induced by surface topography, and the feedback of material deformation on the bubble-collapse process are not considered.

The theoretical calculations presented herein are primarily used to estimate the order of magnitude of the cavitation erosion damage threshold. They are not intended to accurately predict the local impact pressure or spatial energy distribution under the experimental cavitation conditions.

According to the theoretical framework proposed by Zhu [46], the energy associated with a cavitation bubble can be expressed as Eq.(18)

(18) $$E_{B}=\frac{4}{3}R_{B}^{3}\left(P_{d}\;\text{-}\;P_{v}\right)$$

Based on the findings of Xia [47], the average bubble diameter at an ultrasonic frequency of 20 kHz is approximately 20 μm. Since the cavitation frequency used in this study is consistent with that condition, the bubble radius is taken as *R_B_ =* 10 μm. The ambient driving pressure *P_d_* and vapor pressure *P_v_* are adopted from Zhu [46] as 86148.9 Pa and 4979.18 Pa, respectively.Substituting these parameters into Eq. (18), the energy of a single cavitation bubble is estimated to be approximately:*E_B_* = 3.4*e-10J.

According to the study by Lechner [48],the energy released during bubble collapse can be partially converted into the kinetic energy of a microjet. The corresponding energy conversion efficiency can be described by Eq.(19) as follows:

(19) $$\eta_{B}=\max\left(-0.0071\gamma^{2}-0.0293\gamma +0.1513,0\right),0⩽\gamma ⩽3$$

Here, *γ* is determined by the distance between the bubble center and the specimen surface. Since cavitation-induced damage is predominantly governed by microjet impact during bubble collapse, the corresponding energy conversion efficiency becomes a key parameter. Based on the experimental data reported by Lechner [43], when *γ* = 1 the microjet energy conversion efficiency *η_B_* is approximately 15.13%. Therefore, the actual microjet energy can be expressed as:

(20) $$E_{B}^{r}=\eta_{B}*E_{B}$$

According to the assumption proposed by Zhu [46], the characteristic radius of the impact spot is taken as 0.1 times the bubble radius, *rc* = 1 μm. The energy density of the microjet impact spot can therefore be estimated as the energy distributed over the impact area:

(21) $$\Phi =\frac{dE_{B}}{\mathrm{dS}}=\frac{E_{B}^{r}}{\pi r_{c}^{2}}=16.4J/m^{2}$$

When a cavitation bubble collapses near the specimen surface, the resulting microjet impingement generates a transient water hammer pressure, which can be described based on classical impact theory *P* [49] as:

(22) $$P=\rho_{B}c_{B}v_{B}\left(\frac{\rho_{s}c_{s}}{\rho_{B}c_{B}+\rho_{s}c_{s}}\right)$$

Considering the scattering and attenuation of incident pressure waves, Lei [50] established an acoustic attenuation model to refine the classical water hammer formulation. Based on this improvement, the water hammer pressure *P_J_* generated by cavitation bubble collapse can be directly obtained using Eq.(23):

(23) $$P_{J}=8.97c_{B}\left(\frac{\rho_{s}c_{s}}{\rho_{B}c_{B}+\rho_{s}c_{s}}\right)\sqrt{\left(p_{0}+2\pi f\rho_{B}c_{B}Ae^{-\alpha d/2}-p_{v}\right)\rho_{B}}$$

Given that the experimental conditions in Lei [50] are highly comparable to those in the present study, most key parameters were adopted from that work, except for differences in material type and ultrasonic system characteristics. Substituting the relevant parameters into Eq. (23), the microjet-induced water hammer pressure is calculated to be approximately *P_J_* approx 1.2 GPa.

This impact pressure is significantly higher than the yield strength (A) of 1Cr12NiMo2VN steel, indicating that the transient load generated during cavitation bubble collapse is sufficient to induce plastic deformation at the material surface. Under repeated cyclic loading, such high-intensity impacts lead to the accumulation of plastic strain, initiation of microstructural damage, and eventual material removal. Therefore, the high-amplitude impact force generated by cavitation microjets can be regarded as one of the primary mechanical driving mechanisms for WE damage.

The enhancement of WE resistance by UIT can be systematically interpreted through the framework of “energy density–critical threshold–fatigue accumulation.” From the perspective of surface integrity, the untreated rough surface contains numerous micro-scale “peak–valley” features, which serve as preferential sites for bubble attachment and nucleation [41].During WE, the microjet impact associated with bubble collapse is highly localized, leading to “energy focusing” within surface depressions. Coupled with stress concentration effects, this promotes the initiation and propagation of microcracks. As material removal progresses and surface roughness further increases, new nucleation sites continuously form, resulting in a positive feedback loop of “attachment–collapse–damage–reattachment,” thereby accelerating erosion.

**Fig. 25:**
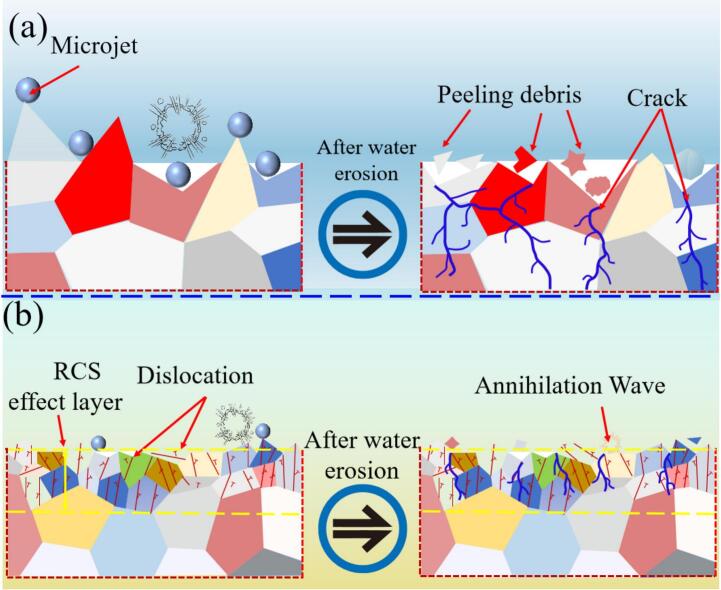
Mechanism of WE performance enhancement.

As illustrated in the mechanism schematic of Fig. 25, after UIT, the surface layer underwent pronounced plastic deformation under high-frequency loading. This process increased the surface hardness, introduced a beneficial RCS field, and improved the surface morphology through a microscale peak-flattening and valley-filling effect[51]. This significantly reduces surface roughness and decreases the number of potential nucleation sites. Fundamentally, this geometric reconstruction mitigates micro-scale stress concentration sources and substantially increases the critical energy density threshold required for the material to resist microjet-induced damage.

Simultaneously, UIT induces a gradient grain distribution within the surface layer of the material. According to the Hall–Petch relationship [52], [53], grain refinement significantly enhances the surface yield strength and strain hardening capacity. This enhancement effectively mitigates plastic loss in the material when subjected to transient impact loads generated by bubble annihilation.From the perspective of fracture mechanics, the action of bubble collapse on the surface is essentially characterized by a transient tensile stress effect. This tensile stress must first counteract the pre-existing RCS before it can effectively drive localized crack propagation. Furthermore, The grain-refined gradient microstructure, increased dislocation density, and possible strain-induced martensitic transformation generated by severe deformation during UIT may act synergistically to enhance the dissipation of impact energy released by cavitation bubble collapse [15], [24], [30], [31], the gradient structure of refined grains and the high-density dislocation networks efficiently dissipate impact energy, thereby elevating the energy threshold for plastic deformation and crack initiation. This synergy further inhibits crack propagation and retards damage evolution.

This microstructural strengthening acts synergistically with the RCS introduced by UIT. The refined and strain-hardened microstructure enhances the intrinsic resistance of the surface layer to deformation and cracking, while the RCS reduces the effective tensile stress generated by repeated cavitation impacts. The combined effects of the modified microstructure and residual stress field delay plastic strain accumulation, crack initiation, and crack propagation, thereby reducing material detachment and improving the WE resistance of 1Cr12Ni3Mo2VN steel. Among the investigated processing conditions, Group B exhibited relatively fine grains, a high level of local misorientation, a high proportion of low-angle grain boundaries, and a continuous gradient-hardened layer, providing a microstructural explanation for its superior WE resistance.

The critical failure energy density of the strengthened material is substantially higher than that of its original state. Consequently, the majority of bubble annihilation events are insufficient to induce immediate damage, shifting the failure mode from “single-impact-dominant transient failure” to “long-term cyclic loading-driven fatigue accumulation.” This transition is the fundamental mechanism behind the significant extension of the WE incubation period: the strengthened surface must undergo high-frequency impact loading to reach the damage threshold, rather than sustaining damage in the initial stage. In summary, UIT achieves a mechanistic shift from “discrete failure” to “cumulative damage” through the synergistic regulation of surface optimization and micromechanical strengthening, thereby remarkably enhancing the WE resistance and service life of the material.

## 5 Conclusion

This study focuses on the water erosion of last-stage steam turbine blades. Using 1Cr12Ni3Mo2VN stainless steel as the research object, ultrasonic impact treatment was employed to enhance its water erosion resistance. To optimize the processing parameters, the effects of static load, vibration amplitude, and coverage on residual stress, surface roughness, and morphology were systematically investigated. Furthermore, the underlying mechanism of UIT in improving cavitation erosion resistance was explored. The main conclusions are as follows:

(1) The effective lateral superposition range of the residual compressive stress induced by UIT was found to be approximately equal to the diameter of a single impact dimple. When the impact spacing exceeded this range, no evident stress-superposition effect was observed. In contrast, surface roughness was mainly controlled by impact coverage, and reducing the impact spacing promoted more uniform plastic deformation and improved surface smoothing. Unlike previous studies that mainly evaluated the overall strengthening effect of ultrasonic surface treatment, the present results distinguishes stress-field regulation from surface-topography regulation and provides a practical criterion for selecting the impact spacing during UIT.

(2) Static load and vibration amplitude jointly determine the magnitude of residual stress, while coverage has a negligible effect on the maximum residual stress. Reaching a relatively high level of either parameter can drive the residual stress into a plateau region, the upper limit of which is determined by the intrinsic properties of the material. However, the synergy between static load and amplitude significantly increases the depth of the residual compressive stress layer.

(3) UIT modification synergistically enhances the WE resistance of 1Cr12Ni3Mo2VN stainless steel by reducing surface roughness and introducing RCS. Cavitation bubble observations indicate that, during the initial stage, the UIT-treated specimens exhibited a lower bubble number density and more localized cavitation activity, suggesting that surface smoothing altered early-stage bubble attachment and heterogeneous cavitation behavior. As WE progressed and the differences in surface roughness gradually diminished, the bubble distributions among the specimens became increasingly similar, indicating that surface morphology was no longer the dominant factor. During the later stage, the RCS and gradient-hardened layer became the primary damage-resistance mechanisms. They mitigated the transient tensile stresses induced by bubble collapse and delayed crack initiation and propagation, thereby governing the improvement in WE resistance. Consequently, the dominant mechanism transitioned from a “roughness-controlled cavitation activity stage” to a “RCS-dominated damage-resistance stage.” Ultrasonic surface treatment generally induces near-surface grain refinement, dislocation accumulation, and crystallographic texture evolution. These microstructural changes may further enhance the resistance to localized plastic deformation and the strain-hardening capacity, while increasing the energy threshold required for crack initiation and propagation. The synergistic regulation of these three factors is the key to achieving enhanced cavitation erosion resistance.

## CRediT authorship contribution statement

**Tong Ran:** Writing – review & editing, Writing – original draft, Software, Data curation, Conceptualization. **Peihan Lin:** Visualization, Validation, Supervision, Software. **Fei Sun:** Writing – review & editing, Supervision, Methodology. **Yongqing Lai:** Writing – review & editing, Writing – original draft, Software, Conceptualization. **YiMing Lin:** Visualization, Validation, Software. **Yu Zhang:** Validation, Supervision, Software. **Yanyu Wang:** Validation, Resources. **Bicheng Guo:** Supervision, Resources, Formal analysis. **Shiqi Chen:** Supervision, Software, Methodology. **Qingshan Jiang:** Writing – review & editing, Validation, Funding acquisition, Formal analysis.

## Declaration of competing interest

The authors declare that they have no known competing financial interests or personal relationships that could have appeared to influence the work reported in this paper.
